
JOURNAL INFORMATION
==============================
NLM Title Abbreviation: Biospektrum (Heidelb)
Journal ID: Biospektrum (Heidelb)
NLM Journal ID: 9615685

Biospektrum

ISSN: 0947-0867
EISSN: 1868-6249

ARTICLE INFORMATION
==============================
PMCID: PMC10101532
PMID: 37073322
DOI: 10.1007/s12268-023-1906-y
Article ID: 1906
Article version: 1
Subjects: Wissenschaft · Special: Molekulare Medizin & Virologie

Molekulare Pinzetten als Breitbandinhibitor viraler Infektionen

Weil Tatjana
Münch Jan jan.muench@uni-ulm.de

grid.410712.1 0000 0004 0473 882X Institut für Molekulare Virologie, Universitätsklinikum Ulm, Meyerhofstraße 1 (ZQB), D-89081 Ulm, Deutschland
Electronic publication date: 2023 Apr 13
Print publication date: 2023
Volume: 29
Issue: 2
First page: 150
Last page: 152
Copyright: © Die Autorinnen und Autoren 2023
License: Open Access: Dieser Artikel wird unter der Creative Commons Namensnennung 4.0 International Lizenz veröffentlicht, welche die Nutzung, Vervielfältigung, Bearbeitung, Verbreitung und Wiedergabe in jeglichem Medium und Format erlaubt, sofern Sie den/die ursprünglichen Autor(en) und die Quelle ordnungsgemäß nennen, einen Link zur Creative Commons Lizenz beifügen und angeben, ob Änderungen vorgenommen wurden. Die in diesem Artikel enthaltenen Bilder und sonstiges Drittmaterial unterliegen ebenfalls der genannten Creative Commons Lizenz, sofern sich aus der Abbildungslegende nichts anderes ergibt. Sofern das betreffende Material nicht unter der genannten Creative Commons Lizenz steht und die betreffende Handlung nicht nach gesetzlichen Vorschriften erlaubt ist, ist für die oben aufgeführten Weiterverwendungen des Materials die Einwilligung des jeweiligen Rechteinhabers einzuholen. Weitere Details zur Lizenz entnehmen Sie bitte der Lizenzinformation auf http://creativecommons.org/licenses/by/4.0/deed.de.
License URL: https://creativecommons.org/licenses/by/4.0/

issue-copyright-statement: © Springer-Verlag GmbH Deutschland, ein Teil von Springer Nature 2023

==============================
The SARS-CoV-2 pandemic once again highlighted the constant threat posed by viruses. Specific therapeutics are highly warranted, but their development is time consuming and cost intensive. Broad-spectrum antivirals provide a promising option for fast application to treat circulating or newly emerged viruses. Here, we introduce molecular tweezers as broad-spectrum antivirals, which abrogate viral infection by directly targeting the viral membrane. Furthermore, we discuss the current stage of tweezer development to fight SARS-CoV-2 and other respiratory viruses.

■ Anhaltende Meldungen über Virusausbrüche und das gehäufte Auftreten von viralen Infektionserkrankungen verdeutlichen die stetige Bedrohung durch Viren. Besonders deutlich wird dies im Kontext von Pandemien: So forderte die Influenzapandemie in den Jahren 1918 bis 1920, bekannt als „Spanische Grippe", 20 bis 50 Millionen Menschenleben [1]. Etwa 100 Jahre später, mit Auftreten des *severe acute respiratory syndrome coronavirus type 2* (SARS-CoV-2) Ende 2019, standen Medizin und Wissenschaft erneut vor einer großen Herausforderung. Die rasche Entwicklung von Medikamenten und vor allem Impfstoffen halfen, die Pandemie einzudämmen. Allerdings starben bis heute über 6,8 Millionen Menschen im Zuge der von SARS-CoV-2 ausgelösten Krankheit COVID-19 (*coronavirus disease 2019*). Die zur Verfügung stehenden Therapeutika bieten dabei einen wirksamen Schutz vor einem schwerem Krankheitsverlauf, jedoch nur bei frühzeitiger Einnahme. Hinzukommt, dass die durch die Impfung produzierten Antikörper nur einen reduzierten Schutz vor Infektion gegen neu auftretenden SARS-CoV-2-Varianten wie Omikron aufweisen [2]. Zusätzlich zeichnen sich manche der neuen Varianten durch eine gesteigerte Übertragbarkeit und eine erhöhte Infektiosität aus [2].

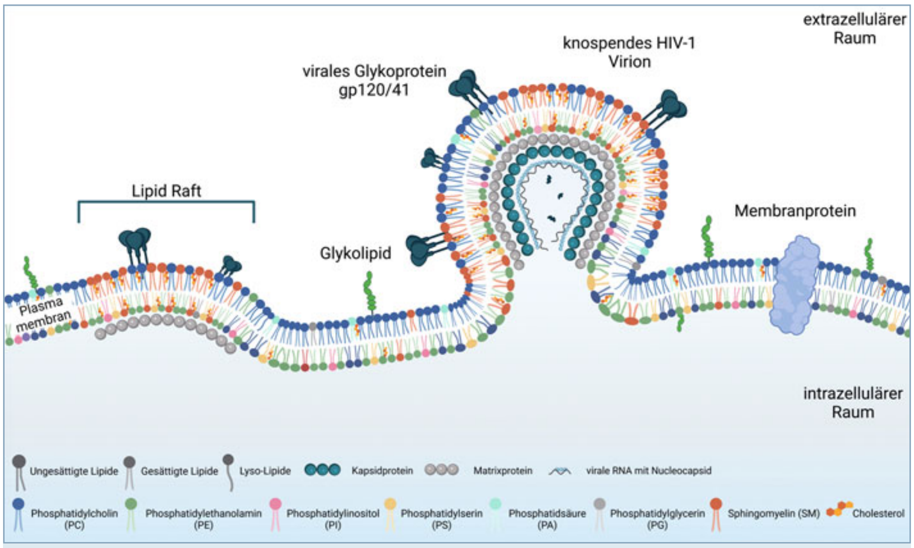

▲ **Abb. 1:** Virale Knospung aus der zellulären Plasmamembran. Die doppellagige Lipidschicht ist heterogen und asymmetrisch aus unterschiedlichen Phospholipiden, Sphingolipiden und Sterinen aufgebaut. Die Knospung von Viren aus der Plasmamembran, wie beispielsweise von HIV-1, erfolgt bevorzugt aus *lipid-rafts*, die angereichert an Sphingomyelin und Cholesterol sind. Erstellt mit BioRender.com.

### Zoonotische Viren als ständige Bedrohung des Menschen

Zoonosen, also Infektionserkrankungen, bei denen eine Übertragung vom Tier zum Menschen stattfindet, sind keineswegs eine Seltenheit. Hierzu zählen auch das Humane Immundefizienz-Virus (HIV-1), Marburg- oder Ebola-Viren, das Zika-Virus, Hanta-Viren und viele weitere. Solche zoonotischen Übertragungen können zu sporadischen, aber auch endemischen oder pandemischen Ausbrüchen führen. Diese Beispiele weisen auf die Dringlichkeit zur „Pandemic Preparedness", der Pandemievorsorge für neuauftretende Erreger hin [3]. Die Entwicklung spezifischer Therapeutika gegen ein Virus oder eine Virusfamilie ist kostspielig, zeitintensiv und setzt Kenntnisse über Replikation und Struktur des Erregers voraus. Daher könnten antivirale Wirkstoffe mit breiter Aktivität eine zunehmend wichtige Rolle in der Pandemievorsorge spielen. Durch gezielten Angriff eines allgemeinen Charakteristikums vieler Viren, wie z. B. der viralen Membran, könnten virale Infektionen umfassend präventiv verhindert oder therapiert werden [3].

### Die virale Membran als Angriffsfläche

Der Großteil der hochpathogenen Viren ist von einer Lipidschicht umgeben, in der für den Eintritt in die Zelle wichtige virale Glykoproteine eingelagert sind. Eine intakte Lipidschicht ist essenziell für die Infektiosität viraler Partikel. Wird diese Membran zerstört, so wird auch das Virus unschädlich gemacht. Viren erhalten ihre Hülle während der Freisetzung – der Knospung – aus der infizierten Zelle (**Abb. 1**). Dabei wird das virale Kapsid von einer zellulären Membran umschlossen. Diese Membran besteht aus einer doppellagigen, heterologen Phospholipidschicht, bei der die hydrophoben Fettschwänze zueinander gerichtet sind, während die hydrophilen Lipidköpfchen nach außen ragen. Die Membran dient der Zelle zur Formgebung, als Schutzbarriere oder zum Transport [4]. Obwohl die Hülle des Virus von der zellulären Membran stammt, unterscheiden sich diese voneinander. Zum einen liegt die Größe eines Viruspartikels im zwei- bis dreistelligen Nanometerbereich, während eine menschliche Zelle mehrere Mikrometer misst. Dadurch hat die virale Membran eine weitaus stärkere Krümmung als die der Zelle. Ein weiterer Unterschied liegt auch in der Lipidzusammensetzung. Die Freisetzung vieler Viren aus der Zelle findet an spezifischen Regionen, den *lipid rafts* statt [5]. Diese Bereiche in der Membran zeichnen sich durch einen hohen Anteil der beiden Lipide Sphingomyelin und Cholesterol aus (**Abb. 1**, [5]). Die Unterschiede in Größe, Krümmung und Zusammensetzung zwischen viraler und zellulärer Membran bieten Angriffspunkte, um gezielt membranumhüllte Viren zu zerstören, ohne die menschliche Zelle zu schädigen.

## Die molekulare Pinzette CLR01 zerstört die virale Membran

Die molekulare Pinzette CLR01 besitzt die Fähigkeit, die Hülle von Viren zu zerstören [6]. CLR01 besteht aus einem elektronenreichen, torusförmigen Kohlenstoffgerüst mit zwei Phosphatgruppen an der zentralen Brücke, und ähnelt einer Pinzette (*tweezer*) (**Abb. 2**). Ursprünglich wurde CLR01 entwickelt, um die Ausbildung pathogener Amyloidfibrillen wie Amyloid β oder α-Synuclein, die bei den Krankheitsbildern Alzheimer und Parkinson eine Rolle spielen, zu unterbinden [7]. Dies erfolgt durch die Bindung der Pinzette an Lysin und Arginin. Beide Aminosäuren befinden sich bei fehlgefalteten Fibrillen frei zugänglich nach außen gerichtet. In späteren Studien konnten wir überraschenderweise zeigen, dass CLR01 auch eine direkte antivirale Aktivität gegen HIV-1 hat [6]. Unsere Untersuchungen zeigten, dass der molekulare *tweezer* die Lipidköpfchen in der viralen Membran umschließt und somit die Orientierung der gebundenen Lipide in der Membran verändert [8]. Dieser Vorgang erlaubt das Eindringen von CLR01 in die Membran, erzeugt eine erhöhte Spannung und letztlich die Zerstörung der Virushülle. CLR01 bindet dabei bevorzugt Sphingomyelin, ein Lipid, welches vermehrt in der Hülle vieler Viren vorkommt. Tatsächlich haben weitere Studien gezeigt, dass CLR01 auch andere aufgetretene umhüllte Viren, wie Zika, Ebola, Herpes, Masern oder Influenza hemmt [8, 9]. Aufgrund der breiten Wirksamkeit ist CLR01 eine vielversprechende Leitstruktur für eine weitere Entwicklung als antivirales Medikament. Allerdings stellte sich heraus, dass Proteine im Blutplasma die antivirale Aktivität von CLR01 neutralisieren [9]. Dies schließt jedoch eine topische Anwendung in den Atemwegen oder im Genitaltrakt zur Prävention oder Therapie respiratorischer Erreger oder sexuell übertragbarer Viren nicht aus.

## Erhöhung der antiviralen Aktivität der Pinzette durch Lipidanker

Mit Beginn der SARS-CoV-2-Pandemie haben wir die Aktivität von CLR01 gegen das neue Coronavirus getestet. Wir konnten zeigen, dass die molekulare Pinzette mit einer Konzentration im höheren mikromolaren Bereich die Infektion durch diesen respiratorischen Erreger verhindert [10]. Um die antivirale Aktivität des Moleküls zu steigern, wurden 34 weitere *tweezer*-Moleküle von der Arbeitsgruppe um Prof. Dr. Thomas Schrader (Universität Duisburg-Essen) synthetisiert. Dabei wurden Alkylketten unterschiedlicher Länge oder aromatische Verbindungen an die Phosphatgruppen von CLR01 gekoppelt, um dabei Komponenten der Membran – wie Fettsäuren oder Steroide – nachzuahmen und das Eindringen der Pinzette in die virale Membran zu steigern. Tatsächlich hemmten Pinzetten mit Hexyl- und Heptyl- oder aromatischen Seitenketten eine SARS-CoV-2-Infektion mit nanomolarer Aktivität [10]. Computergestützte Simulationen und weitere Experimente zeigten, dass der Mechanismus zur

Hier steht eine Anzeige.
Springer

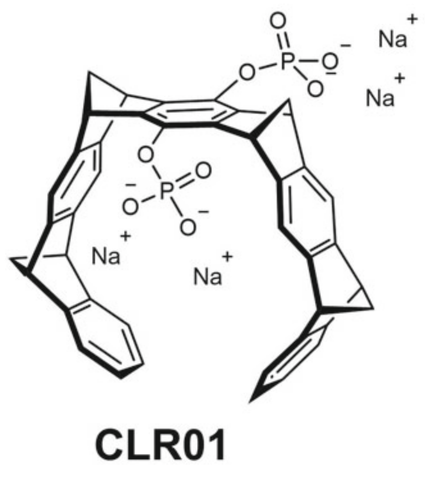

▲ **Abb. 2:** Die molekulare Pinzette CLR01. CLR01 besteht aus einem torusförmigen Kohlenstoffgerüst mit zwei Phosphatgruppen in der Mitte. Abbildung wurde erstellt von Thomas Schrader; Universität Duisburg-Essen, aus JACS Au 2022; 2: 2187–2202, https://doi.org/10.1021/jacsau.2c00220; © 2022 *The Authors*, Lizenz CC BY-NC-ND 4.0; Erlaubnis zur Publikation wurde beim Ersteller eingeholt.

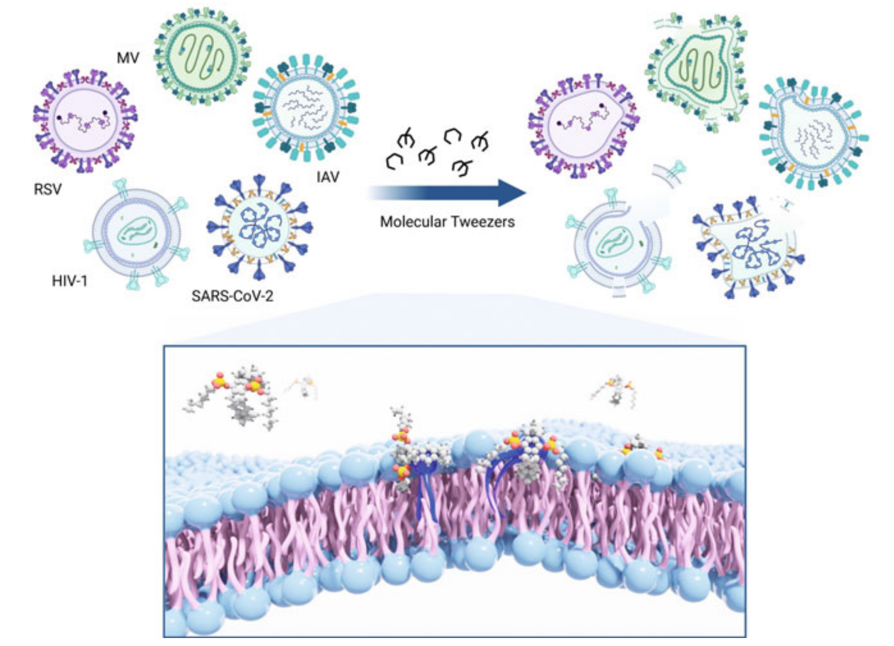

▲ **Abb. 3:** Molekulare Pinzette als Breitbandinhibitor viraler Infektionen. Die molekulare Pinzette umschließt ein Lipidköpfchen der viralen Membran und verändert dessen Ausrichtung. Der Prozess erlaubt das Eindringen der Pinzette in die Membran, wodurch sich die Spannung erhöht und die Viruslyse induziert wird. Pinzetten mit Seitenketten können diese zusätzlich in die Membran inkorporieren, was eine Steigerung der antiviralen Aktivität zur Folge hat. MEV: Masernvirus; RSV: Respiratorisches Synzytial-Virus; IAV: Influenza-A-Virus; SARS-CoV-2: *Severe acute respiratory syndrome coronavirus type 2*; HIV-1: Humanes Immundefizienz-Virus
Abbildung wurde erstellt von Tatjana Weil, Lukas Wettstein und Joel Mieres Pérez; Universität Ulm und Duisburg-Essen, aus [10], © 2022 *The Authors*, Lizenz CC BY-NC-ND 4.0; Erlaubnis zur Publikation wurde beim Ersteller eingeholt.

Membranzerstörung erhalten bleibt und die *tweezer* der zweiten Generation zusätzlich fähig sind, ihre Seitenketten in die virale Membran zu inkorporieren (**Abb. 3**). Dies erhöht die Oberflächenspannung rascher und ermöglicht eine schnellere Lyse der Viruspartikel. Diese optimierten *tweezer*-Derivate inaktivierten wie erwartet auch andere umhüllte Erreger, wie das Masernvirus (MEV), das Respiratorische Synzytial-Virus (RSV), Influenza-A-Virus (IAV), oder HIV-1 (**Abb. 3**) und stellen einen vielversprechenden antiviralen Breitbandwirkstoff dar, welcher gute Aussichten für die klinische Entwicklung zur Bekämpfung neu auftretender respiratorischer Viren hat. ■


Funding note: Open Access funding enabled and organized by Projekt DEAL.

Tatjana Weil 2013–2019 Biologiestudium an der Universität Ulm. Seit 2019 Promotion am Institut für Molekulare Virologie am Universitätsklinikum Ulm.

Jan Münch 1993–1998 Biologiestudium an der Universität Erlangen mit dort anschließender Promo tion bis 2002. Von 2002–2004 Postdoktorand am Institut für Virologie am Universitätsklinikum Ulm mit daraufhin folgender W1-Professur. Im Jahr 2010 Wechsel an das Institut für Molekulare Virologie in Ulm als W3-Professor und seit 2017 dortiger Co-Direktor.


Literatur

[1] Trilla A Trilla G Daer C The 1918 “Spanish Flu” in Spain Clinical Infectious Diseases 2008 47 668 673 10.1086/590567 18652556
[2] Chi WY Li YD Huang HC COVID-19 vaccine update: vaccine effectiveness, SARS-CoV-2 variants, boosters, adverse effects, and immune correlates of protection J Biomed Sci 2022 29 82 10.1186/s12929-022-00853-8 36243868 PMC9569411
[3] Yoon BK Jeon WY Sut TN Stopping Membrane-Enveloped Viruses with Nanotechnology Strategies: Toward Antiviral Drug Development and Pandemic Preparedness ACS Nano 2021 15 125 148 10.1021/acsnano.0c07489 33306354
[4] Lombard J Once upon a time the cell membranes: 175 years of cell boundary research Biol Direct 2014 9 32 10.1186/s13062-014-0032-7 25522740 PMC4304622
[5] Sezgin E Levental I Mayor S The mystery of membrane organization: composition, regulation and roles of lipid rafts Nat Rev Mol Cell Biol 2017 18 361 374 10.1038/nrm.2017.16 28356571 PMC5500228
[6] Lump E Castellano LM Meier C A molecular tweezer antagonizes seminal amyloids and HIV infection eLife 2015 4 e05397 10.7554/eLife.05397 26284498 PMC4536748
[7] Shahpasand-Kroner H Siddique I Malik R Molecular tweezers — supramolecular hosts with broad-spectrum biological applications Pharmacol Rev 2023 75 263 308 10.1124/pharmrev.122.000654 36549866 PMC9976797
[8] Weil T Groß R Röcker A Supramolecular Mechanism of Viral Envelope Disruption by Molecular Tweezers J Am Chem Soc 2020 142 17024 17038 10.1021/jacs.0c06400 32926779 PMC7523239
[9] Röcker AE Müller JA Dietzel E The molecular tweezer CLR01 inhibits Ebola and Zika virus infection Antiviral Research 2018 152 26 35 10.1016/j.antiviral.2018.02.003 29428508 PMC7113745
[10] Weil T Kirupakaran A Le MH Advanced Molecular Tweezers with Lipid Anchors against SARS-CoV-2 and Other Respiratory Viruses JACS Au 2022 2 2187 2202 10.1021/jacsau.2c00220 36186568 PMC9516563
